# Monthly Summary

**Published:** 1868-06

**Authors:** 


### MONTHLY SUMMARY.

---

*A Monster Tumor.*—On Wednesday last the theatre of University College Hospital was literally crammed from top to bottom by visitors from all parts of London who had come to see Mr. Christopher Heath remove an unusually large tumor of the lower jaw. The patient was a man, thirty-two years of age, whose appearance was most extraordinary from the enormous enlargement of the lower part of his face. An idea of this may be gained by mention of the fact that a line taken round the chin, from one ear to the other, measured nineteen and a half in.; and from the lower lip (which was dragged down) to the pomum Adami, thirteen in. Mr. Heath made a vertical cut through the lower lip at its centre, extended it nearly to the thyroid cartilage, reflected the flaps, sawed through the body of the left jaw just behind the second molar tooth, disarticulated the head of the right jaw, and removed, in this way, a mass of osteo-sarcoma weighing 4 lb. 8 oz. The operation was performed with singular expedition and facility, and but very little blood was lost. Mr. Heath was assisted by Mr. Erichsen, Sir Henry Thompson, Mr. Marshall, and Mr. Berkeley Hill, who illustrated, it seemed to us, exactly what good assistance should be. Mr. Clover managed the chloroform (in spite of the great difficulties of the case) so cleverly that the patient was kept under its influence throughout the operation. It may be noted that the tumor is the largest of its kind on record since Mr. Syme took one away, of the same weight in 1828.—*Lancet.*

*On the Causes of Alopecia and of its Greater Frequency in Males than Females.*—Dr. A. F. King, after referring to the statement of Wilson (*On Diseases of the Skin,*) that the cause of alopecia occurring in persons of advanced years was to be "sought for in an impediment to circulation through the textures of the scalp of the upper part of the head," presented some remarks endeavouring to establish the proposition *that the impediment to the circulation* referred to *was due to compression—either partial or complete—of the arteries supplying the scalp by pressure of tight-fitting hats.* The vessels were compressed, and, consequently, the amount of blood flowing through them diminished by the impinging of a hard hat-rim upon the resisting protuberances of the cranium.

In support of this proposition Dr. King mainly relied upon the greater frequency with which the disease occurred in males than females, hats being worn as a general rule only by the former. Those worn by women did not come low enough on the sides of the head to compress the arteries, but rather rested on the top secured by strings. Moreover, men being more in the open air wore their hats during a longer period than females. If the idea of Wilson was correct that this difference between the sexes depended upon a larger quantity of adipose tissue being situated beneath the integument of the scalp in the female, whereby a more easy and unimpeded transit was afforded for the minute vessels to the capillary plexus of the derma, it might still be a question whether the extra amount of fat *itself* in women was not dependent upon the same freedom of circulation in the scalp which secured them a better supply of hair.

Dr. K. next referred to Fig. 209 of *Gray's Anatomy* (2d Am. edit., p. 374,) and showed how a line drawn across it, representing the hat-rim, would strike the branches of the anterior temporal arteries over the frontal protuberances, the region where the hat pressure was most severe. It would also compress the posterior temporals at or near the parietal ridges, and the occipitals in the same way behind. The reason why baldness occurred in different localities in different individuals was, probably, due to a difference in the shape of the head. A long skull, where the hat pressure would be most exerted on the forehead (and, consequently, on the anterior temporal arteries) and occiput, would loose its hair soonest from the top and anterior portion; and, probably, also to some extent low down behind from pressure of the occipital artery. This last vessel, however, does not contribute very much to the circulation on the top of the scalp. When a patch of baldness exists about the vertex we might expect to find a *wide* head, the posterior temporal arteries, which supply the locality referred to, being compressed against the parietal bones. The little tuft or island of hair so often observed, left by itself on the top of the forehead, was nourished by the two supra-orbital arteries, these vessels having escaped hat pressure by passing over the forehead in the slight concavity between the two frontal eminences.

To say that the pressure of a tight-fitting hat exerts *no* influence upon capillary circulation in the scalp is unreasonable. In the absence of a bandage, in a case of hemorrhage from a wounded branch of the temporal artery, how could we better arrest the bleeding than by placing on the patient a close-fitting hat, a compress being adjusted under its rim immediately over the trunk of the vessel!

Below the hat-rim, where the circulation is not impeded, we invariably find the hair to remain good, though the top of the head may, at the same time, be entirely bald.

In many cases of alopecia where all recognized causes of the disease are from the circumstances of the case excluded, there is no other plausible way of accounting for the disease than by the deranged circulation occasioned by pressure upon the arteries.

In conclusion, Dr. K .recommended as a prophylactic measure the manufacture of hats made so as to embrace the head at points or surfaces where no considerable vessel would be compressed, or hats made to order for each individual might be arranged with a notch or semi-circular concavity in the rim over the spot of skin under which the artery passed.

If the anatomy of the part was explained to intelligent hat-makers, it is probable some one of them might devise a modification of the present form of hat which would be less destructive to the hair.

The cost of such an innovation to consumers would hardly exceed that annually expended in the purchase of quack nostrums in the form of "hair restoratives."—*Proc. Clin. Path. Soc. of Washington D. C., N, O. Jour. Med.*

---

"*With Brains, Sir.*"—Professor Tiedemann assumed that, inasmuch as a certain size and mass of brain was essential for the exercise of the faculties of mind, all human races are furnished therewith in an equal degree. Dr. Barnard Davis, in an elaborate paper read last week before the Royal Society, analyzed the existing evidence, and added extensive additional observations which lead to a different result. The average brain-weight for the English is stated to be 47.50 ounces; of the French, 44.58 ounces; of the German, 42.83 ounces, (but there are discrepancies in the results of different observers.) The Italians, Lapps, Swedes, Frisians, and Dutch come into the same category with the English. Among the Asiatic races, the Vedahs of Ceylon and the Hindoos give a mean of over 42.11 ounces. The skulls of Mussulmans afford a slightly increased average of brain-weight over those of Hindoos. Two skulls of male Khonds, one of the unquestioned aboriginal races of India, show a brain-weight of only 37.87 ounces. The general average of the Asiatic table shows a diminution of more than two ounces when compared with the Europeans. The general mean of African races is less than that of European races, according to Dr. Davis, although there are great differences, the Kaffir rising high and the Bushman sinking low in the scale. The average of the whole of the aboriginal American races reaches 44.73 ounces, which is 2.13 ounces less than that of the European races. The Australian races show a brain-weight one-ninth less than that of the general average of Europeans. The Malays and other of the "Oceanic races" who migrated boldly for commercial purposes over the North and South Pacific Oceans and occupy the islands show a tolerably high average of brain-weight, and on arriving at this section we seem to be returning in some measure to the large brain-weight of Europeans.—*Medical Gazette.*

---

*Keratitis from Inherited Syphilis, wihout Peculiarities of Physiognomy or Teeth.*.—In this case a girl aged seven, was brought with keratitis, which Mr. Hutchison diagnosed as of the interstitial or syphilitic form. Her physiognomy was, however, not peculiar, and her teeth were of good size and form. Her mother gave the history of suspicious symptoms in infancy, and stated that the child was under the care of Dr. Dobdell for them. On application to him, Dr. Dobdell was courteous enough to send a report of the child's illness, fully confirming the diagnosis. He had attended both the mother and child for syphilis. This conclusive testimony was very valuable in the absence of the usual physiognomical peculiarities. We may perhaps go further, and suggest that the careful treatment (by mercury) which the child had in infancy has perhaps been the means of much diminishing the severity of the disease, and thus preventing the production of the characteristic physiognomy and teeth.

In this case, as well as in a preceding, in which the teeth were characteristically malformed, the attack of keratitis has been less severe than usual, and in each it has fallen chiefly on only one eye. In the latter case the treatment has been by the iodide of potassium, two grains three times daily.—*London Med. Times & Gazette.*

---

*External Use of Arnica.*—As an accident resulting from the *use of arnica externally*, in cases of bruises, wounds, falls, ect., Drs. Gallasse and Mazzoni, of Rome, describe an eruption resembling that resulting from frictions with croton oil, with redness and swelling, very much like erysipelas, and accompanied with fever.— *Union Medical,*

---

